# Editorial: Innovative approaches to foster healthy cultures and mental health in sport

**DOI:** 10.3389/fspor.2023.1209370

**Published:** 2023-05-15

**Authors:** Eric E. Hall, Paul Anthony Davis, Natalie Durand-Bush, Jade Salim

**Affiliations:** ^1^ Department of Exercise Science, Elon University, Elon, NC, United States; ^2^ Department of Psychology, Umeå University, Umeå, Sweden; ^3^ School of Human Kinetics, University of Ottawa, Ottawa, ON, Canada; ^4^ Department of Sport and Exercise Science, St Mary's University, Twickenham, United Kingdom

**Keywords:** mental health, sports, cultural differences, positive psychology, well-being

**Editorial on the Research Topic** Innovative approaches to foster healthy cultures and mental health in sport

There is increasing interest and prioritization of mental health in sport. This has resulted in numerous position stands by leading sporting organizations that draw attention to the importance of athlete mental health for both well-being and performance. However, the environment plays an important role and it is essential to expand existing views and approaches regarding athlete mental health to address the impact of other leaders and factors within the sports environment.

The aim of this research topic was to highlight innovative and comprehensive approaches to research and applied practice serving to optimize mental health in sport while increasing our understanding of the environment. In our initial call, we recognized the need for “multi and interdisciplinary approaches or systems to foster mental health and safe sport environments.” One paper that demonstrated this was the article by McCabe et al. who examined perspectives regarding the integration of mental health and nutrition within Division I university athletics programs. To this end, they interviewed 17 athletic personnel—including athletic trainers, coaches, dietitians, sport psychologists, strength and conditioning coaches, and sports medicine physicians. Four themes emerged: (1) Resources (e.g., monetary and staffing), (2) Education (e.g., need for more knowledge for all in sports departments to support), (3) Department Integration or Collaboration (e.g., communication between different members of the organization), and (4) Student and Coach Engagement (e.g., support of coaches is important for athlete utilization of resources). Overall, this novel study highlights the value of consolidating mental health and nutrition and supports the need for an environment in which all parties have a role in supporting mental health.

An increasing body of research highlights that an athlete's social environment can influence perceptions of burnout through antecedents as well as protective mechanisms (1); however, there is limited understanding of the impact of an athlete's contextual social interactions on the potential manifestation of burnout. As such, Appleby et al. outline a preliminary validation of the Teammate Burnout Questionnaire (TBQ) as a measure of athletes' perceptions of their teammates' burnout. Their study reports satisfactory discriminant and convergent validity of the established Athlete burnout questionnaire (2) and the newly developed TBQ as a measure of burnout at the team level. The availability of the validated TBQ can contribute to the study of team culture and its implications for athlete mental health and burnout. More specifically, future research may examine the mechanisms underpinning the development of burnout within a team by examining the role of interpersonal communication and emotion regulation. Increased understanding can promote more effective expressions of emotion and interactions between teammates to foster healthy cultures and mental health.

An important leader in creating a positive and healthy cultural climate is the coach, and to be fully able to foster this type of environment, it is necessary for them to be mentally healthy. In the paper by Wright et al., they examined whether burnout was a mediator between stress (e.g., perceived and workplace) and mental health in a study of collegiate coaches in the U.S. The authors found that burnout (as measured by the Oldenburg Burnout Inventory) was not a strong predictor of mental health (measured through the Depression, Anxiety, and Stress Scale and Warwick-Edinburgh Mental Well-being Scale). While disengagement, one of the burnout scales, was a predictor of depression and well-being, the most consistent predictors of mental health were perceived life stressors. Additionally, workplace stressors were significantly related to burnout. While the results of this study should be taken with the COVID-19 pandemic in consideration, it does suggest the importance of proper interventions to create a mentally healthy environment in which all can succeed.

Interventions may be provided in different forms when fostering mental health and it is important to recognize the various types of practitioners who contribute to this. In the U.S. and Canada, Mental Performance Consultants (MPCs) typically help athletes, coaches, and staff to manage and improve their performance and well-being, as well as their highly dynamic training and competition environment. When individuals experience more serious mental health challenges and mental illness symptoms, practitioners such as clinical psychologists, counselors, psychotherapists, and social workers provide support, with or without collaboration with MPCs. Collaborative care is considered a best practice in mental health care delivery and it has seldom been studied in the context of sport. In the paper by Van Slingerland et al., the authors present a novel interdisciplinary approach used within the Canadian Centre for Mental Health and Sport to provide mental health care to athletes. Using an illustrative case study approach, the authors demonstrate the interactions that occurred between mental performance and mental health practitioners to provide care to a high-performance athlete over 11 months, as well as factors facilitating and impeding the team's collaboration. The collaborative process was primarily driven by the client's complex symptoms and needs, which varied across time. Facilitative factors included the secure online platform and tools, as well as individual and team characteristics such as communication, trust, support, and shared workload. Collaboration was, however, hindered by logistical challenges, overlapping scopes of practice, and the client's over-reliance on one particular practitioner. Overall, there were more perceived benefits than drawbacks to providing collaborative care, and practitioners' willingness to work together to establish and monitor care plans was deemed essential for success.

In summary, the aim of this research topic was to highlight novel studies advancing our knowledge and capacity to improve mental health and sport environments. The four papers highlight different variables and factors that contribute to mental health experiences and outcomes in sport. A common thread throughout the four papers is inter/multi-disciplinary work via collaboration. For example, given evidence linking mental health and nutrition, collaboration between practitioners appears to be valuable to integrate both of these variables when designing and implementing comprehensive interventions. Furthermore, while burnout has traditionally been examined at an individual level, there is merit in assessing its different dimensions at a team level using the Teammate Burnout Questionnaire (TBQ). Collaboration amongst teammates can not only help to prevent burnout but also lead to timely interventions when teammates perceive that a peer is showing signs and symptoms of this syndrome. Stress and burnout are inter-related variables directly or indirectly impacting mental health in sport. Collaboration between practitioners and coaches is important so that coaches are capable of managing the stressors they encounter in their environment. This can avert the experience of burnout and mental illness symptoms such as anxiety and depression. Finally, interdisciplinary work between different types of practitioners such as MPCs and clinical psychologists, counselors, psychotherapists, and social workers can lead to the design and delivery of optimal mental health care plans for athletes.

In terms of future directions, research should continue to explore variables and collaborative frameworks to improve mental health and psychologically healthy and safe environments in sport. An important focus that should be prioritized in future studies is the interplay between mental health, mental illness, mental performance, and sport culture. Given the continuing rise in safe sport and social justice issues in the sport environment, it is imperative that researchers and practitioners collaborate to find approaches and solutions to enhance sport experiences and outcomes at all levels. Mental health promotion and mental illness prevention have never been more important, and strengthening mental performance and cultural competencies in athletes, coaches, leaders, and staff may be a vital way to address this. Collaboration will be essential to make sport healthy and safe for all!
